# Scorpion venom as a source of new antimicrobial agents

**DOI:** 10.3389/fmicb.2026.1816327

**Published:** 2026-09-07

**Authors:** Yuanyuan Zhang, Rongqian Wu

**Affiliations:** 1 Department of Pediatrics, The First Affiliated Hospital of Xi'an Jiaotong University, Xi'an, China; 2 National Local Joint Engineering Research Center for Precision Surgery and Regenerative Medicine, Shaanxi Provincial Center for Regenerative Medicine and Surgical Engineering, The First Affiliated Hospital of Xi’an Jiaotong University, Xi'an, China

**Keywords:** antimicrobial peptide, mechanism, pathogenic microorganism, scorpion venom, therapeutics

## Abstract

The escalating global threat of antimicrobial resistance necessitates urgent discovery of novel therapeutic agents. Scorpion venom, a complex cocktail of bioactive molecules, has emerged as a promising reservoir of antimicrobial peptides (AMPs) with potent activity against multidrug-resistant pathogens. This review comprehensively examines the structural and functional diversity of scorpion-derived AMPs and their therapeutic potential. Scorpion venom AMPs exhibit remarkable structural heterogeneity. Their physicochemical properties, characterized by cationic charge, amphipathicity, and α-helical or β-sheet conformations, underpin their antimicrobial efficacy. These peptides demonstrate broad-spectrum antimicrobial activity encompassing Gram-positive and Gram-negative bacteria, fungi, viruses, and parasites. Mechanistically, scorpion AMPs employ multifaceted strategies including membrane disruption through pore formation or carpet-like mechanisms, cell wall synthesis inhibition, intracellular interference with DNA replication and protein synthesis, biofilm dispersion, immunomodulation, and ion channel modulation. This polypharmacological approach has been proposed to lower the risk for resistance development relative to conventional antibiotics, though this remains to be comprehensively validated across diverse scorpion AMPs. Despite their therapeutic promise, challenges persist regarding cytotoxicity, proteolytic instability, and high production costs. However, rational engineering strategies, including sequence truncation, D-amino acid substitution, cyclization, and hybrid peptide design, are advancing clinical viability. Notable candidates have demonstrated exceptional potency in preclinical models. This review synthesizes current knowledge on scorpion venom AMPs, highlighting their advantages over traditional antibiotics and outlining optimization strategies. Future research directions emphasize structure–activity relationship studies, novel delivery systems, and comprehensive toxicological profiling. Scorpion venom represents an underexploited natural resource that may yield next-generation antimicrobial therapeutics critical for combating the growing threat of drug-resistant infections.

## 1 Introduction

The inappropriate and excessive use of conventional antibiotics has precipitated a global health crisis characterized by the rapid emergence of multidrug-resistant (MDR) pathogens (Patel et al., 2024; Gajic et al., 2025; Macesic et al., 2025; Wolford et al., 2025). Microorganisms belonging to the ESKAPE group, *Enterococcus faecium*, *Staphylococcus aureus*, *Klebsiella pneumoniae*, *Acinetobacter baumannii*, *Pseudomonas aeruginosa*, and *Enterobacter* species, pose particularly significant threats due to their increasing resistance to available antibiotics (Hetta et al., 2025; Rabea et al., 2025). This alarming trend, coupled with the declining pipeline of new antimicrobial agents, underscores the urgent need for innovative therapeutic strategies (Primon-Barros and Macedo, 2017; Bartalucci et al., 2025).

Animal venoms, refined through millions of years of evolutionary selection, represent a rich and largely unexploited source of bioactive molecules with therapeutic potential (Almaaytah and Albalas, 2014; Sani and Separovic, 2016; Oliveira Júnior et al., 2025; Yang et al., 2025). Among venomous organisms, scorpions have garnered particular attention due to the remarkable diversity of peptides present in their venoms (Abdallnasser Amen et al., 2025; Dahiya et al., 2026). These arachnids have inhabited the Earth for over 400 million years, during which their venom arsenals have evolved to effectively immobilize prey and deter predators (Rates et al., 2011). The resulting molecular diversity encompasses peptides with varied structures and functions, including numerous antimicrobial peptides (AMPs) that protect the scorpion itself from pathogenic microorganisms (Ranasinghe and McManus, 2013).

Scorpion venom AMPs offer several advantages over conventional antibiotics, including broad-spectrum activity, rapid bactericidal action, and a lower propensity for inducing resistance. Unlike traditional antibiotics that typically target specific bacterial proteins or metabolic pathways, many AMPs exert their effects mainly through membrane disruption, a mechanism that is more difficult for bacteria to circumvent (Peter Muiruri et al., 2023). Several recent reviews have summarized scorpion-derived AMP research (Almaaytah and Albalas, 2014; Xia et al., 2024; Xin et al., 2025). Distinct from these prior reviews, the present work emphasizes the polypharmacological multi-mode action of scorpion AMPs and the experimental evidence gaps for resistance-reduction hypotheses; it further provides systematic comparative analysis of native peptides versus rationally engineered analogs, deep translational discussion covering ADMET profiling, multi-omics-AI-assisted peptide discovery, synthetic-biology production, nanoparticle formulation, combination therapy and personalized infection-treatment perspectives. This review provides a comprehensive examination of scorpion venom as a source of new antimicrobial agents, encompassing the structural and functional diversity of these peptides, their mechanisms of action, spectrum of antimicrobial activity, and prospects for clinical development.

## 2 Structural diversity of scorpion venom antimicrobial peptides

### 2.1 Classification based on disulfide bridge content

Scorpion venom peptides are broadly classified into two major categories based on the presence or absence of disulfide bridges: disulfide-bridged peptides (DBPs) and non-disulfide-bridged peptides (NDBPs) (Remijsen et al., 2010; Panayi et al., 2024).

Disulfide-bridged peptides (DBPs) typically contain three or four disulfide bonds that confer structural stability and define their three-dimensional conformation (Ranasinghe and McManus, 2013). These peptides primarily function as ion channel modulators, targeting voltage-gated sodium, potassium, and calcium channels (Ortiz et al., 2015; Xin et al., 2025). While their primary role is neurotoxic, some DBPs also exhibit antimicrobial activity, suggesting functional multifunctionality (Ranasinghe and McManus, 2013; Abdallnasser Amen et al., 2025).

Non-disulfide-bridged peptides (NDBPs) constitute a structurally diverse group characterized by the absence of cysteine residues and disulfide bonds (Zeng et al., 2005; Remijsen et al., 2010). These linear peptides typically range from 13 to 50 amino acids in length and display considerable sequence variability (Zeng et al., 2005; Panayi et al., 2024). NDBPs are the primary source of antimicrobial activity in scorpion venoms, although some also exhibit anticancer, immunomodulatory, and bradykinin-potentiating activities (Zeng et al., 2005; Remijsen et al., 2010). Based on sequence similarity and pharmacological functions, NDBPs have been classified into several subfamilies, with antimicrobial peptides representing one of the most prominent groups (Zeng et al., 2005).

### 2.2 Physicochemical properties of scorpion AMPs

The antimicrobial activity of scorpion venom peptides is governed by several key physicochemical properties that determine their mechanism of action and therapeutic potential (Fong-Coronado et al., 2024; Panayi et al., 2024). A core structural principle governing the antimicrobial activity of scorpion venom-derived AMPs is amphipathicity, defined as the spatial segregation of amino acid residues into distinct hydrophobic and hydrophilic faces within the peptide structure. This fundamental structural feature drives the organization of scorpion AMPs into conformations with discrete faces: one face composed of hydrophobic residues and the opposing face consisting of hydrophilic residues. This spatial segregation is not only a defining characteristic of scorpion AMPs but also serves as the foundation for their functional activity, mediating key interactions with target membranes and underpinning their antimicrobial mechanisms (Harrison et al., 2014; Fong-Coronado et al., 2024; Kim et al., 2026). The hydrophobic character of peptides influences their ability to interact with and insert into lipid bilayers. Optimal hydrophobicity is required for membrane insertion, while excessive hydrophobicity can lead to aggregation and reduced solubility (Fong-Coronado et al., 2024). Key parameters quantifying these properties include the hydrophobic moment, which measures amphipathicity, and the grand average of hydropathy (GRAVY) value, which reflects overall hydrophobicity (Fong-Coronado et al., 2024).

Amphipathicity is closely intertwined with the cationic nature of scorpion AMPs, which is essential for their initial electrostatic interaction with negatively charged microbial membranes. Most scorpion AMPs carry net positive charges typically ranging from +1 to +8 at physiological pH (Fong-Coronado et al., 2024; Panayi et al., 2024). This positive charge facilitates electrostatic interaction with the negatively charged components of bacterial membranes, including lipopolysaccharides (LPS) in Gram-negative bacteria and lipoteichoic acids (LTA) in Gram-positive bacteria (Ranasinghe and McManus, 2013; Harrison et al., 2014). Peptides with higher net positive charges generally exhibit stronger antimicrobial activity, although excessive charge can increase hemolytic activity against mammalian cells. The optimization of net charge represents an important strategy for improving the therapeutic index of scorpion venom peptides. Additionally, C-terminal amidation, frequently observed in short scorpion AMPs (typically 13–25 amino acids), enhances stability and antimicrobial activity (Fong-Coronado et al., 2024; Panayi et al., 2024). Beyond neutralizing negative charges and increasing overall net positive electrostatic charge, C-terminal amidation can induce critical conformational changes: it remodels peptide flexibility, modulates amphipathic secondary-structure propensity, and reshapes peptide-membrane interaction behavior, as exemplified by scorpion peptides Uy234 and AamAP1-Lys. Furthermore, amidation confers direct proteolytic protection by resisting carboxypeptidase-driven C-terminal degradation, improving peptide persistence within biological fluids. Collectively, these electrostatic, conformational and proteo-stabilizing effects jointly tune antimicrobial potency and therapeutic index.

The propensity to form alpha-helical structures upon membrane interaction is a key determinant of antimicrobial activity for many scorpion venom peptides (Amorim-Carmo et al., 2022). Structural studies employing circular dichroism and nuclear magnetic resonance spectroscopy have revealed that many scorpion AMPs adopt α-helical conformations upon interaction with membrane environments, although β-sheet and extended structures are also observed (Amorim-Carmo et al., 2022). Peptides that readily adopt amphipathic alpha-helical conformations typically exhibit potent membrane-disrupting properties (Amorim-Carmo et al., 2022). Structural modifications that enhance alpha-helical content can improve antimicrobial activity, as demonstrated by engineered analogs of natural scorpion peptides (Möller et al., 2025). Intrinsic flexibility also plays a crucial role, influencing the dynamics of membrane interactions and overall antimicrobial potency (Amorim-Carmo et al., 2022).

These interconnected physicochemical properties, cationic charge, amphipathicity, secondary structure propensity, and conformational flexibility, collectively underpin the antimicrobial efficacy of scorpion venom peptides and provide rational guidelines for their therapeutic optimization.

### 2.3 Species-specific diversity

Scorpion venom production is a conserved trait across the entire Order Scorpiones, which comprises more than 2,900 valid scorpion species globally (The Scorpion Files, NTNU. Available online: https://www.ntnu.no/ub/scorpion-files/). Individual scorpion venoms can contain hundreds of bioactive peptides; to date only approximately 400 scorpion-venom peptides have been biochemically characterized (Solano-Godoy et al., 2025). Despite this vast unexplored potential, characterized peptides often share similarities in primary and tertiary structures as well as genomic organization. This reflects a common evolutionary pattern where protein superfamilies conserve a structural scaffold while diversifying biochemical and biological functions (Zhijian et al., 2006).

Nevertheless, the antimicrobial peptide repertoire varies considerably across scorpion species, reflecting their diverse ecological niches and evolutionary histories (Rates et al., 2011). The family *Buthidae*, which includes medically significant genera such as *Androctonus*, *Leiurus*, *Mesobuthus*, and *Tityus*, has been particularly well-studied for AMP content (Anandhan Sujatha et al., 2024; Wiezel et al., 2024; Lafnoune et al., 2025).

The genus *Androctonus*, prevalent in North Africa and the Middle East, produces approximately 24 distinct AMPs with demonstrated antimicrobial, antifungal, and antiproliferative activities (Anandhan Sujatha et al., 2024). Species such as *A. australis*, *A. crassicauda*, and *A. mauritanicus* have yielded peptides with potent activity against both Gram-positive and Gram-negative bacteria (Li et al., 2019; Anandhan Sujatha et al., 2024).

The South American genus *Tityus*, responsible for most scorpion accidents in Brazil, produces venoms rich in antimicrobial peptides alongside neurotoxins (Yacoub et al., 2020; Wiezel et al., 2024). Transcriptomic analysis of *T. stigmurus* venom glands has revealed diverse components, including antimicrobial peptides with therapeutic potential (Yacoub et al., 2020). Similarly, the Asian scorpion *Buthus martensii Karsch* (BmK) has been extensively studied, yielding numerous peptides with antimicrobial, anticancer, and analgesic properties (Rates et al., 2011; Mosaheb et al., 2018).

Other genera, including *Pandinus*, *Heterometrus*, and *Parabuthus*, have also contributed notable antimicrobial peptides (Zeng et al., 2005; Perumal Samy et al., 2017). Parabutoporin from *Parabuthus schlechteri* exemplifies a multifunctional peptide with antimicrobial activity at micromolar concentrations and immunomodulatory effects at submicromolar concentrations (Zeng et al., 2005).

## 3 Spectrum of antimicrobial activity

### 3.1 Antibacterial activity

Scorpion venom peptides demonstrate potent antibacterial activity against a wide range of clinically relevant pathogens, including both Gram-positive and Gram-negative bacteria (Rincón-Cortés et al., 2022; Rabea et al., 2025). Among Gram-positive bacteria, scorpion AMPs effectively inhibit *Staphylococcus aureus*, including methicillin-resistant *S. aureus* (MRSA), one of the most problematic nosocomial pathogens (Primon-Barros and Macedo, 2017; Song et al., 2022; Fong-Coronado et al., 2024). Peptides such as meucin 24 and meucin 25 from *Mesobuthus eupeus* exhibit potent activity against *S. aureus* with minimal inhibitory concentrations (MICs) in the micromolar range (Perumal Samy et al., 2017). Activity against other Gram-positive pathogens, including *Enterococcus faecalis*, *Bacillus subtilis*, and *Streptococcus* species, has also been documented (Bhavya et al., 2016; Rincón-Cortés et al., 2022). These peptides also show remarkable efficacy against Gram-negative pathogens, including multidrug-resistant members of the ESKAPE group such as *Pseudomonas aeruginosa*, *Acinetobacter baumannii*, and *Klebsiella pneumoniae* (Fong-Coronado et al., 2024; Rabea et al., 2025), owing to their ability to disrupt both outer and inner membranes (Harrison et al., 2014).

Representative examples from Table 1 illustrate key Structure–activity relationship (SAR)-to-toxicity trade-offs. BmKn2-7 (derived from *Mesobuthus martensii)* shows potent dual-Gram-positive/Gram-negative activity (MIC 3.13 μg/mL against *S. aureus*, 6.25 μg/mL against *E. coli*); its higher net positive charge (+3) compared with parent BmKn2 (+1) improves potency but still retains measurable hemolysis (50% at 90.27 μg/mL). Hp1404 from *Heterometrus petersii* preferentially targets *Gram-negative* bacteria, a relatively rare trait among short-chain NDBPs, though it displays considerable hemolytic liability (50% hemolysis at 226.6 μg/mL). Stigmurin (derived from *Tityus stigmurus*) acts mainly against Gram-positive bacteria; amino-acid substitutions generating StigA6-StigA31 increase net charge, broaden spectrum toward Gram-negative strains, yet simultaneously elevate hemolytic risk. Many native scorpion AMPs have higher MIC values relative to clinical antibiotics such as vancomycin; nevertheless, rationally-engineered analogs achieve drastically improved potency against MDR-ESKAPE pathogens. Conserved sequence hallmarks across these antibacterial candidates include frequent cationic Lys/Arg residues, N-terminal hydrophobic residues, amphipathic α-helix-forming propensity and frequent C-terminal amidation for short variants. A consistent SAR trade-off is observed: elevated hydrophobicity enhances antibacterial potency but frequently aggravates hemolytic damage toward mammalian host cells. A few examples of the scorpion-venom-derived antibacterial peptides are presented in Table 1.

**Table 1:** Scorpion venom-derived antibacterial peptides.

Name	Source Scorpion	Sequence	Post-translational modification	Physicochemical properties (% of α-helix)	Minimal inhibitory concentrations (MIC)	Hemolytic activity	References
BmKn2-7	*Buthus martensii Karsch*	FIKRIARLLRKIF	C-terminal amidation	100	*S. aureus* 3.13 μg/mL *E. coli* 6.25 μg/mL	50% hemolysis at 90.27 μg/mL	Cao et al. (2012), Nishihara et al. (2024)
Ctriporin	*Chaerrilus tricostatus*	FLWGLIPGAISAVTSLIKK	C-terminal amidation	73.6	*S. aureus* 5 μg/mL *E. coli* >100 μg/mL	90.6% hemolysis at 256 μg/mL	Fan et al. (2011), Amorim-Carmo et al. (2022), Luo et al. (2024)
Hp1404	*Heterometrus petersii*	GILGKLWEGVKSIF	C-terminal amidation	Not specified	*S. aureus* 6.25–12.5 μg/mL *E. coli* >100 μg/mL	50% hemolysis at 226.6 μg/mL	Li et al. (2014), Hong et al. (2021)
Imcroporin	*Isometrus maculates*	FFSLLPSLIGGLVSAIK	C-terminal amidation	100%	*S. aureus* 20 μg/mL *E. coli* >100 μg/mL	< 50% hemolysis at 50 μg/mL	Zhao et al. (2009), Sahoo et al. (2022)
meucin-18	*Mesobuthus martensii*	FFGHLFKLATKIIPSLFQ	No	76.4	*S. aureus* 8 μg/mL *E. coli* 128 μg/mL	37.7% hemolysis at 6.25 μM	Amorim-Carmo et al. (2022), Chang et al. (2025)
Pepcon	Consensus design*	FSLIPSAIGGLISA	Likely C-terminal amidation	Not specified	*S. aureus* 5 μg/mL *E. coli* 50 μg/mL	< 40% hemolysis at 100 μM	Almaaytah et al. (2017)
Stigmurin	*Tityus stigmurus*	FFSLIPSLVGGLISAFK	C-terminal amidation	58.8	*S. aureus* 8.68 μM *E. coli* >139 μM	22% hemolysis at 139.5 μM	de Melo et al. (2015), Amorim-Carmo et al. (2022)
TsAP-1	*Tityus serrulatus*	FLSLIPSLVGGSISAFK	C-terminal amidation	58.82	*S. aureus* 120 μM *E. coli* 160 μM	6.48% hemolysis at 160 μM	Guo et al. (2013), Amorim-Carmo et al. (2022)
TsAP-2	*Tityus serrulatus*	FLGMIPGLIGGLISAFK	C-terminal amidation	76.47	*S. aureus* 5 μM *E. coli* >320 μM	86% hemolysis at 40 μM	Guo et al. (2013), Amorim-Carmo et al. (2022)

*Pepcon was designed as a consensus peptide derived from multiple sequence alignment of a highly homologous group of scorpion antimicrobial peptides.

Furthermore, emerging evidence indicates that scorpion venom peptides possess activity against mycobacterial species, including *Mycobacterium tuberculosis* and non-*tuberculous mycobacteria* (Memariani and Memariani, 2025), acting through multiple mechanisms such as plasma membrane ATPase inhibition, membrane depolarization, cell wall disruption, and induction of reactive oxygen species (Memariani and Memariani, 2025). Notably, in murine models, administration of venom-derived peptides at doses ≤2 mg/kg body weight reduced mycobacterial burden and inflammation, demonstrating dual therapeutic and immunomodulatory effects (Memariani and Memariani, 2025).

### 3.2 Antifungal activity

A growing body of research indicates that several scorpion venom peptides exhibit significant antifungal activity against a spectrum of clinically important fungal pathogens, offering a promising alternative to conventional treatments. Prominent species from the *genera Candida*, *Cryptococcus*, and *Aspergillus* are particularly susceptible to these scorpion AMPs, which often target fundamental cellular structures; specifically, they typically act through direct membrane disruption, frequently exploiting the higher affinity for fungal ergosterol over mammalian cholesterol to ensure selectivity, and the induction of lethal levels of reactive oxygen species that damage intracellular components (Rincón-Cortés et al., 2022).

The clinical relevance of these findings is underscored by the increasing incidence of invasive fungal infections, especially among immunocompromised populations such as those undergoing chemotherapy or living with HIV, coupled with the limited arsenal of antifungal agents currently available and the rising prevalence of azole-resistant strains (Rincón-Cortés et al., 2022). Furthermore, existing antifungal therapies often suffer from significant host toxicity, such as nephrotoxicity associated with polyenes, highlighting the need for safer alternatives. Consequently, the unique physicochemical properties, potent efficacy, and potential for synthetic optimization of scorpion-derived antifungal peptides make them particularly attractive candidates for therapeutic development, potentially filling critical gaps in the management of multidrug-resistant mycoses and reducing the global burden of fungal disease (Rincón-Cortés et al., 2022).

Table 2 summarizes eight antifungal scorpion peptides from six independent publications. These candidates fall into non-disulfide-bridged peptides (NDBPs) and disulfide-bridged peptides (DBPs). NDBPs primarily exert antifungal effects via ergosterol-dependent fungal-membrane permeabilization and ROS-triggered intracellular damage; DBP peptide Ts1 mediates antifungal activity via putative ion-channel modulation. Several peptides display low-micromolar *in vitro* antifungal potency comparable to first-line antifungal agents, yet a large proportion carry substantial hemolytic risk. GK-19 stands out as a favorable native lead combining strong antifungal activity and negligible hemolysis. Sequence-wise, short-chain NDBP antifungal peptides commonly adopt amphipathic α-helical structures; long-chain NDBPs show higher sequence divergence, while DBP antifungal peptides operate via distinct structural scaffolds unrelated to typical amphipathic helical motifs. These natural antifungal peptides remain pre-clinical leads; rational sequence modification is necessary to optimize selectivity.

**Table 2:** Scorpion venom-derived antifungal peptides.

Name	Source scorpion	Sequence	Target fungi and MIC	Hemolytic activity	References
Pantinin-1	*Pandinus imperator*	GILGKLWEGFKSIV	*C. tropicalis* 16 μM	21% hemolysis at 64 μM	Zeng et al. (2013)
Pantinin-2	*Pandinus imperator*	IFGAIWKGISSLL	*C. tropicalis* 16 μM	81% hemolysis at 32 μM, 100% at 64 μM	Zeng et al. (2013)
Pantinin-3	*Pandinus imperator*	FLSTIWNGIKSLL	*C. tropicalis* 17 μM	70% hemolysis at 16 μM, 100% at 32 μM	Zeng et al. (2013)
GK-19	*Androctonus amoreuxi*	GFLFKLIPKAIKKLISKFK	*C. albicans* 10 μM, *C. krusei* 5 μM, *C. glabrata* 10 μM	negligible hemolytic activity at 100 μM	Song et al. (2022)
ToAP2	*Tityus obscurus*	FFGTLFKLGSKLIPGVMKLFSKKKER	*C. tropicalis* 3.12 μM, *C. neoformans* 6.25–12.5 μM, *C. albicans* 12.5 μM	50% hemolysis at 25 μM	Guilhelmelli et al. (2016)
Parabutoporin	*Parabuthus schlechteri*	FKLGSFLKKAWKSKLAKKLRAKGKEMLKDYAKGLLEGGSEEVPGQ	*N. crassa* 2.5 μM, *B. cinerea* 3.5 μM, *F. culmorum* 0.3 μM, *S. cerevisiae* 2 μM	50% hemolysis at 38 μM	Moerman et al. (2002), Rincón-Cortés et al. (2022)
Opistoporin 1	*Opistophtalmus carinatus*	GKVWDWIKSTAKKLWNSEPVKELKNTALNAAKNLVAEKIGATPS	*N. crassa* 0.8 μM, *B. cinerea* 3.1 μM, *F. culmorum* 0.8 μM, *S. cerevisiae* 2 μM	30% hemolysis at 100 μM	Moerman et al. (2002), Rincón-Cortés et al. (2022)
Ts1	*Tityus serrulatus*	KEGYLMDHEG CKLSCFIRPS GYCGRECGIK KGSSGYCAWP ACYCYGLPNW VKVWDRATNKC	*A. nidulans* 2.18 μM	non-cytolytic on erythrocytes at 8.72 μM	Santussi et al. (2017)

NDBP, Non-disulfide-bridged peptide; DBP, Disulfide-bridged peptide.

### 3.3 Antiviral activity

Accumulating evidence demonstrates that scorpion venom peptides possess broad-spectrum antiviral activity against diverse virus families, representing a promising frontier in virology and drug discovery (Albuquerque and Meneses, 2021; El Hidan et al., 2021). Significant antiviral effects have been documented against arboviruses, including dengue, Zika, yellow fever, West Nile, and chikungunya viruses, which pose severe global health threats (El Hidan et al., 2021; Amorim-Carmo et al., 2022). These scorpion peptides inhibit viral replication through mechanisms, such as interfering with viral entry, membrane fusion, intracellular replication, and subsequent release, effectively halting the infection cycle (Albuquerque and Meneses, 2021; Amorim-Carmo et al., 2022). Beyond arboviruses, activity against hepatitis viruses has been reported, with venom components showing therapeutic promise against both HBV and HCV infections, addressing chronic liver disease burdens (Keikha et al., 2022).

Furthermore, the emergence of SARS-CoV-2 has renewed interest in antiviral peptides, positioning scorpion venoms as sources of anti-coronavirus agents capable of addressing pandemic challenges (El Hidan et al., 2021). Structurally, the high isoelectric point and the abundance of basic amino acids, particularly lysine, correlate with enhanced antiviral activity, facilitating electrostatic interactions with negatively charged viral envelopes or host receptors to prevent attachment (Amorim-Carmo et al., 2022). This combination of efficacy and structural properties makes scorpion-derived peptides attractive candidates for developing next-generation antiviral therapeutics to combat resistant strains and fill gaps where vaccines remain unavailable, offering hope for managing viral outbreaks.

Table 3 collects representative antiviral scorpion-venom-derived peptides with diverse mechanisms: viral envelope permeabilization, blockade of viral entry, modulation of host interferon-mediated immune responses, and interference against viral polymerases or spike-host receptor interactions. Some peptides exhibit promising in-vitro IC50 values, but most candidates lack rigorous *in vivo* validation. Several peptides show host-cell cytotoxicity at higher working concentrations, restricting systemic-use prospects. Sequence-conserved traits include high isoelectric points and enrichment of basic Lys- and Arg-rich residues, which enable electrostatic attraction to negatively charged viral envelopes. These are early-stage *in vitro* findings; further optimization of stability, selectivity and pharmacokinetic profiles is mandatory for therapeutic development.

**Table 3:** Scorpion venom-derived antiviral peptides.

Name	Source scorpion	Target virus and IC50	Cytotoxic activity	Mechanism of action	References
Ctry2459	*Chaerilus tryznai*	HCV 1.84 μg/mL	CC50 at 79.8 μg/mL on Huh7.5.1 cells	Destabilize the viral structural integrity	Hong et al. (2013), Raj et al. (2025)
Hp1090	*Heterometrus petersii*	HCV 5.0 μM	Minimally cytotoxic to Huh7.5.1 cells at 20 μg/mL	Permeabilize the viral envelope	Yan et al. (2011), Rincón-Cortés et al. (2022)
Hp1036	*Heterometrus petersii*	HSV-1 0.43 μM	CC50 at 46.71 μM on Vero cells	Inhibit the replication cycle	Hong et al. (2014), Rincón-Cortés et al. (2022)
Mucroporin-M1	*Lychas mucronatus*	Measles 3.52 μM, SARS-CoV 7.12 μM and H5N1 1.03 μM	CC50 at 34.70 μM on Vero cells	Binding to virus membranes; Against the Heptad Repeat 1 domain and RNA-dependent RNA polymerase	Li et al. (2011), Rincón-Cortés et al. (2022), Souza et al. (2023)
BmKn2–7	*Buthus martensii Karsch*	HIV 2.76 μg/mL	CC50 at 38.46 μg/m on TZM-bl cells	Binding to virus membranes	Chen et al. (2012), Wang and Wang (2016)
Smp76	*Scorpio maurus palmatus*	HCV 0.01 μg/mL, dengue virus 0.01 μg/mL	CC50 > 10 μg/m on Huh7it-1 cells	Targeting the viral envelope and upregulating IFN-β	Ji et al. (2018), El-Bitar et al. (2020), Lima et al. (2022)
AM29A5syn	*Androctonus amoreuxi*	SARS-CoV-2200 nM	No evidence of cytotoxic to A549-hACE2 cells at 1.6 mM	Inhibit SARS-CoV-2 spike protein interaction with hACE2 receptor and additional unexplored modes of action	Ghazal et al. (2024)
Ev37	*Euscorpiops validus*	Reduce Dengue virus (91%), HCV (97%) and ZIKV (87%) at 10 μm	CC50 at 116.3 μm on Huh7 cells	Restrict viral late entry by alkalizing acidic organelles	Li et al. (2019), Lima et al. (2022)

### 3.4 Antiparasitic activity

Scorpion venoms and their antimicrobial peptides exhibit promising activity against various parasites, including Plasmodium species responsible for malaria, a disease that continues to cause significant morbidity and mortality worldwide (Salimo et al., 2023). A comprehensive review identified 50 isolated substances, 4 venom fractions, and 7 venom extracts from various animals, including scorpions, with demonstrable antimalarial activity, highlighting the therapeutic potential of these natural compounds (Salimo et al., 2023). These bioactive molecules act at different stages of the Plasmodium life cycle, inhibiting key processes essential for parasite survival, such as erythrocyte invasion, intraerythrocytic development, and gametocyte formation, thereby disrupting transmission and disease progression (Cao et al., 2014; Salimo et al., 2023). Beyond malaria, antiparasitic activity extends to other clinically significant protozoan parasites, including Leishmania species responsible for cutaneous and visceral leishmaniasis, and Trypanosoma species that cause Chagas disease and African sleeping sickness (Pimentel et al., 2021; Soltan-Alinejad et al., 2025).

The multifunctional nature of scorpion AMPs, which combines direct antiparasitic effects, such as membrane permeabilization, mitochondrial dysfunction, and induction of apoptotic-like death, with immunomodulatory activity that enhances host defense responses, makes them particularly attractive candidates for treating neglected parasitic diseases that disproportionately affect developing countries with limited healthcare resources (Cao et al., 2014). Furthermore, their ability to overcome drug resistance mechanisms that compromise current therapies, coupled with their potential for synthetic optimization to improve stability and bioavailability, positions scorpion-derived peptides as valuable leads for developing novel, affordable, and effective antiparasitic agents capable of addressing the global burden of these devastating infections.

## 4 Mechanisms of antimicrobial action

**Figure 1:**
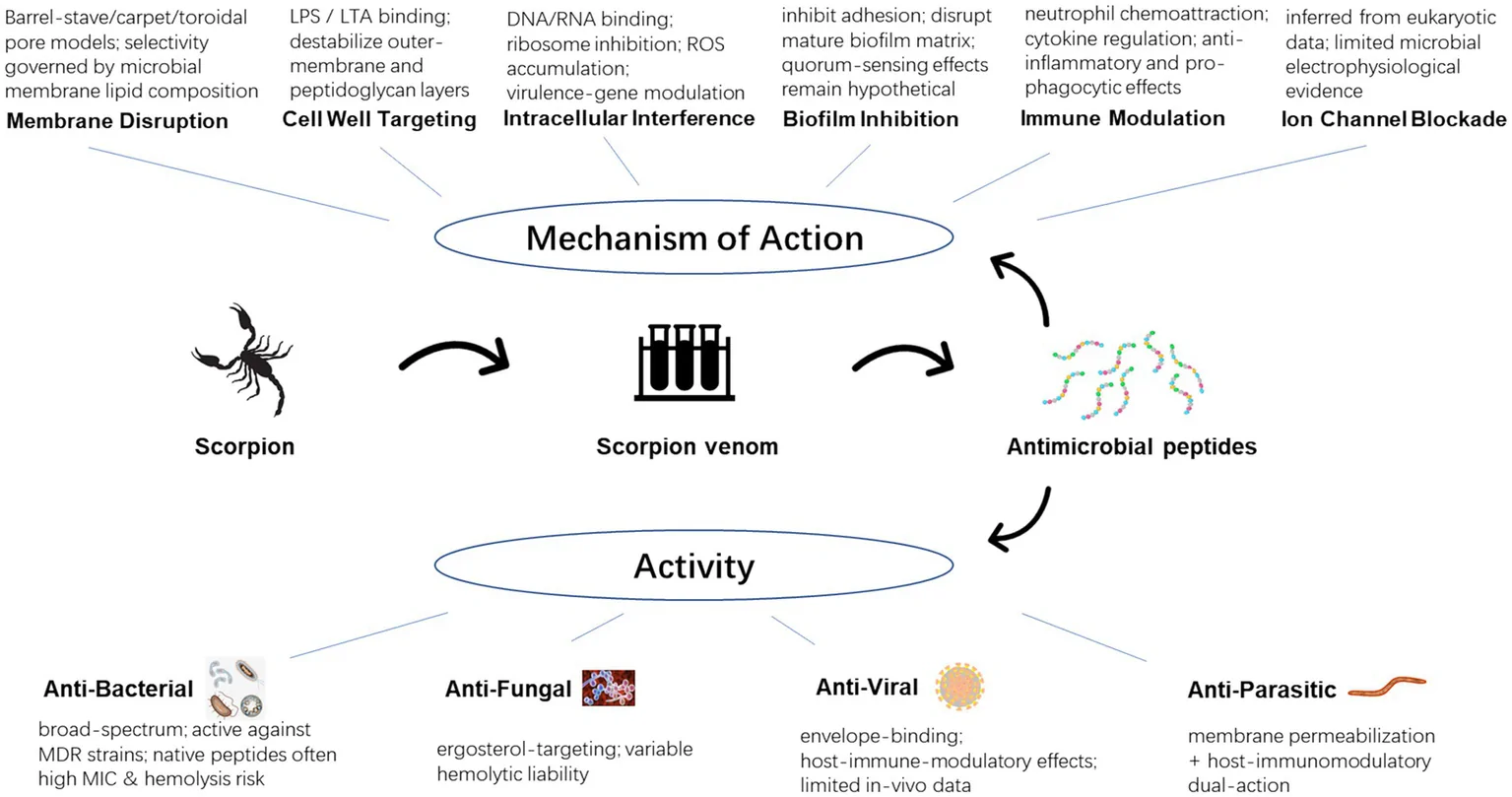
Schematic overview of antimicrobial mechanisms and biological activities of scorpion-venom-derived antimicrobial peptides. This schematic integrates evidence synthesized within this review, summarizing the workflow from scorpion venom to antimicrobial peptides, multi-mode mechanisms of action, and corresponding biological activities. Mechanisms include membrane disruption, cell-wall targeting, intracellular interference, biofilm inhibition, immunomodulation, and ion-channel blockade. Note that ion-channel-blockade effects on microbial cells are largely inferred from eukaryotic-system electrophysiology, with limited direct microbial experimental validation. Some proposed mechanisms (e.g., scorpion-AMP-dependent quorum-sensing interference) still await direct experimental confirmation. Representative supporting evidence is derived from Ranasinghe and McManus (2013), Harrison et al. (2014), Xia et al. (2024). Biological outputs cover antibacterial, antifungal, antiviral and antiparasitic functions. Native scorpion AMPs display broad-spectrum potency *in vitro* yet face multiple translational bottlenecks including cytotoxicity, proteolytic instability and sub-optimal pharmacokinetic profiles.

Scorpion venom-derived antimicrobial peptides represent a promising class of natural compounds with potent activity against a broad spectrum of pathogenic microorganisms, including bacteria, fungi, viruses, and parasites. These peptides employ diverse mechanisms of antimicrobial action that extend beyond simple membrane disruption to include cell wall targeting, intracellular pathway interference, biofilm inhibition, immune modulation, and ion channel blockade (Figure 1).

### 4.1 Membrane disruption

The disruption of microbial membrane integrity represents the primary and most extensively studied mechanism of antimicrobial action for scorpion venom peptides (Kourie and Shorthouse, 2000; Harrison et al., 2014). This process typically occurs through several distinct but interconnected stages, each contributing to the ultimate permeabilization of the target cell membrane and subsequent cell death. The initial stage is characterized by electrostatic interactions between the cationic scorpion AMPs and the negatively charged microbial membranes (Ranasinghe and McManus, 2013; Harrison et al., 2014). The cationic nature, conferred by positively charged residues such as lysine, arginine, and histidine, promotes selective binding. This selectivity is further enhanced by the distinct lipid composition of microbial membranes, containing acidic phospholipids such as phosphatidylglycerol, cardiolipin, and phosphatidylserine in bacteria, and phosphatidylinositol in mycobacteria. Conversely, mammalian membranes comprise zwitterionic phospholipids such as phosphatidylcholine, phosphatidylethanolamine, and sphingomyelin, along with significant cholesterol. Cholesterol contributes to membrane rigidity and reduces peptide affinity for host cells, enhancing selective toxicity toward pathogens. This differential binding forms the basis for therapeutic selectivity, targeting pathogens while minimizing host tissue damage.

Upon contact, scorpion AMPs undergo structural transitions critical for activity. In aqueous solution, many exist as unstructured or partially structured random coils. However, within the lipid bilayer, peptides adopt well-defined amphipathic secondary structures, most commonly alpha-helical conformations (Harrison et al., 2014). The amphipathic alpha-helical structure positions hydrophobic and hydrophilic residues on opposing faces, facilitating optimal bilayer interaction. Hydrophobic faces interact with acyl chains, while hydrophilic faces orient toward the aqueous environment. Driven by the hydrophobic effect, this rearrangement is essential for insertion. Circular dichroism and nuclear magnetic resonance spectroscopy studies show peptides like Pandinin 2 assume alpha-helical conformations in membrane-mimetic environments (Corzo et al., 2001; Ranasinghe and McManus, 2013). Similarly, NDBP-5.5 adopts an amphipathic alpha-helix upon interaction (Trentini et al., 2017).

Following membrane accumulation and conformational change, AMPs insert into the bilayer, disrupting the normal organization of the membrane (Ranasinghe and McManus, 2013). Several mechanistic models have been proposed to explain the pore formation process (Ranasinghe and McManus, 2013). In the barrel-stave model, peptides assemble into transmembrane pores through oligomerization. The carpet model proposes peptides coat the surface until a threshold concentration triggers micellization and disintegration. The toroidal model involves peptides inducing lipid monolayer bending, creating pores lined by peptide and lipid head groups. Regardless of the specific pore formation mechanism, permeabilization leads to detrimental effects ultimately resulting in cell death. Consequences include leakage of cellular contents, dissipation of gradients essential for ATP synthesis and nutrient transport, ATPase inhibition, and reactive oxygen species generation causing oxidative damage to cellular components (Kourie and Shorthouse, 2000).

### 4.2 Cell Wall targeting

Beyond the primary mechanism of membrane disruption, scorpion venom peptides employ a crucial alternative strategy by directly targeting components of the microbial cell wall (Cao et al., 2012). This architectural barrier is vital for structural integrity and protection against osmotic pressure; thus, its compromise significantly threatens cell viability and offers a secondary line of defense against resistant strains. As antibiotic resistance escalates, targeting the cell wall provides a distinct advantage because these structures are less prone to the rapid mutations that affect protein-based drug targets.

In Gram-negative bacteria, lipopolysaccharides (LPS) within the outer membrane serve as key targets. LPS comprises lipid A, a core oligosaccharide, and the O-antigen polysaccharide. The negatively charged phosphate groups in lipid A attract cationic peptides through strong electrostatic forces, often initiating a “self-promoted uptake” pathway (Scott et al., 2000). This binding frequently displaces stabilizing divalent cations like magnesium and calcium, destabilizing the outer membrane. For instance, Kn2-7, an analog derived from BmKn2 of Mesobuthus martensii, disrupts *Escherichia coli* cell walls by binding directly to LPS (Cao et al., 2012). This interaction not only weakens the outer membrane but also aids peptide translocation to internal targets. Additionally, binding to LPS can neutralize the endotoxic activity of lipid A, offering therapeutic advantages against Gram-negative infections by potentially mitigating septic shock symptoms associated with endotoxin release during bacterial lysis (Wang et al., 2020).

In Gram-positive bacteria, lipoteichoic acids (LTA) are the primary anionic targets. Anchored in the cytoplasmic membrane and extending through the thick peptidoglycan layer, LTA maintains cell wall integrity and cation homeostasis. The negatively charged phosphate groups of LTA bind cationic peptides similarly to LPS. Kn2-7 also disrupts *Staphylococcus aureus* cell walls via LTA binding, compromising the envelope and facilitating access to the cytoplasmic membrane (Cao et al., 2012). This mechanism ensures potent activity against resistant pathogens like MRSA, where traditional antibiotics often fail due to modified penicillin-binding proteins. By targeting the cell wall directly, these peptides bypass such resistance mechanisms and may also inhibit biofilm formation, which is often mediated by teichoic acids (Ahn et al., 2018).

Furthermore, activity extends to mycobacteria, which possess complex, mycolic acid-rich cell walls that are notoriously impermeable to standard drugs. Polydim-I, a 22-amino acid peptide from the venom of *Polybia dimorpha*, induces morphological alterations and compromises *Mycobacterium abscessus* cell wall integrity (das Neves et al., 2016). Scanning electron microscopy confirms that such peptides cause visible structural damage, leading to altered morphology and reduced viability. This direct cell wall targeting is particularly valuable for overcoming the unique defensive structures of mycobacteria, suggesting that venom-derived peptides could serve as potent adjuvants or standalone therapies for difficult-to-treat infections like tuberculosis (Carcamo-Noriega et al., 2019). Ultimately, the ability to disrupt both membranes and cell walls highlights the multifaceted therapeutic potential of venom peptides, reducing the likelihood of resistance development while enhancing bacterial clearance in complex clinical scenarios. Such multi-targeting approaches are essential for staying ahead of evolving microbial defense mechanisms.

### 4.3 Intracellular interference

Beyond their effects on cellular membranes and cell walls, certain scorpion venom peptides can penetrate microbial cells and interfere with essential intracellular processes (Memariani and Memariani, 2025). Following translocation across the compromised membrane, these peptides engage in a sophisticated multi-target assault that cripples bacterial physiology from within, a feature less common in conventional antibiotics. For certain characterized peptides such as Smp43, intracellular localization can interfere with nucleic-acid-dependent processes, potentially via direct binding to DNA or RNA, thereby interfering with critical transcription and translation processes required for replication and growth (Tawfik et al., 2021). Furthermore, for select characterized scorpion AMPs, specific peptides inhibit ribosomal function to halt protein synthesis and disrupt cell division by interfering with septum formation, effectively preventing bacterial proliferation. Similarly, individual peptide candidates can target essential bacterial enzymes involved in cell wall synthesis and metabolic pathways, dismantling the organism’s metabolic infrastructure and energy production (Memariani and Memariani, 2025).

A prime example of this dual functionality is Smp43, derived from the Egyptian scorpion *Scorpio maurus palmatus*. This peptide not only compromises bacterial cell membranes but also penetrates the cell to interfere with intracellular gene expression (Tawfik et al., 2021). By modulating gene expression, Smp43 affects the production of essential proteins, stress responses, and virulence factors, thereby compromising bacterial survival and reducing pathogenicity beyond simple cell lysis (Tawfik et al., 2021). Additionally, scorpion venom AMPs induce oxidative stress responses within bacterial cells (Tawfik et al., 2021; Zeng et al., 2025). Treatment with certain peptides leads to increased generation of reactive oxygen species (ROS), causing widespread oxidative damage to cellular components, including lipid peroxidation, protein denaturation, and nucleic acid degradation (Li et al., 2025a, 2025b). This stress is further evidenced by the induction of siderophore biosynthesis, reflecting the activation of bacterial stress responses as the cell attempts to cope with the peptide assault and induced iron scarcity (Tawfik et al., 2021).

Collectively, this multi-target mechanism contributes to a remarkably low propensity for resistance development. For individual multi-targeting scorpion AMPs, the evolutionary burden for bacteria to acquire simultaneous mutations across multiple distinct targets may substantially restrict resistance emergence, though this scenario requires further empirical testing (Utkin et al., 2022). Because the peptides attack multiple distinct physiological pathways simultaneously, the evolutionary burden on the bacteria is immense. This complex mode of action, combining membrane disruption, intracellular interference, and oxidative stress induction, underscores the potential of scorpion venom peptides as robust therapeutic agents capable of overcoming traditional resistance mechanisms inherent in conventional antibiotics. Consequently, the synergy between physical membrane damage and biochemical intracellular inhibition creates a lethal environment that bacteria struggle to evade, ensuring sustained efficacy in clinical applications.

### 4.4 Biofilm inhibition

Bacterial biofilms represent structured communities of bacterial cells enclosed in a self-produced polymeric matrix that are adherent to inert or living surfaces. Biofilm formation significantly enhances bacterial resistance to antimicrobial agents and host immune responses, making biofilm-associated infections particularly difficult to treat. Scorpion venom peptides have demonstrated significant potential in inhibiting biofilm formation and disrupting established biofilms (Capasso et al., 2025; Luo et al., 2025).

Certain scorpion venom peptides can inhibit biofilm formation without necessarily killing planktonic bacteria. BmKn-22, a modified peptide derived from the parental BmKn-2 scorpion venom peptide, has been shown to specifically inhibit biofilm formation by *Pseudomonas aeruginosa* without exhibiting significant bactericidal activity against planktonic cells (Teerapo et al., 2019). This inhibitory effect is dose-dependent with almost 50% inhibition at 800 μM against the formation of *P. aeruginosa* biofilms. This selective anti-biofilm activity provides a new direction for developing antibacterial agents with unique modes of action that differ from conventional antibiotics. Similarly, Hp1404, identified from the venom of the scorpion *Heterometrus petersii*, has demonstrated specific activity against biofilm formation from multidrug-resistant *Pseudomonas aeruginosa* strains, with minimal biofilm inhibitory concentration (MBIC) values ranging from 6.25–50 μM (Kim et al., 2018). This mechanism may involve interference with quorum sensing systems, inhibition of extracellular polymeric substance production, or prevention of initial bacterial adhesion to surfaces.

In addition to preventing biofilm formation, certain scorpion venom peptides can disrupt established biofilms. The amphipathic nature of these peptides allows them to penetrate the biofilm matrix and interact with bacterial cells embedded within the biofilm structure (Yuan et al., 2022; Capasso et al., 2025). By disrupting the extracellular matrix and killing embedded bacterial cells, scorpion venom peptides can effectively eradicate established biofilms, addressing a major challenge in the treatment of chronic infections.

### 4.5 Immune modulation

Beyond their direct antimicrobial activity, scorpion venom peptides modulate host immune responses, enhancing pathogen clearance while potentially reducing inflammation-associated tissue damage (Zeng et al., 2005; Abdallnasser Amen et al., 2025). This immunomodulatory activity represents a unique mechanism that distinguishes scorpion venom peptides from conventional antibiotics, as they enhance the host’s inherent ability to combat infections rather than solely targeting the pathogen. Specific peptides exemplify this multifunctionality; for instance, Parabutoporin acts as a chemoattractant for neutrophils, inducing degranulation, delaying neutrophil apoptosis, and inhibiting superoxide production at submicromolar concentrations (Zeng et al., 2005). These immunomodulatory effects are mediated through interference with intracellular signaling pathways, including G-protein-coupled receptor signaling and calcium mobilization (Zeng et al., 2005). Similarly, BmKbpp, a novel cationic and alpha-helical peptide from the Chinese scorpion *Mesobuthus martensii*, acts as a signaling molecule involving innate immune regulation at low concentrations (Zeng et al., 2012). This peptide modulates various aspects of the innate immune response, including the activation of immune cells and the production of inflammatory mediators, thereby contributing to the clearance of infectious pathogens.

Other scorpion AMPs have demonstrated the ability to modulate cytokine production, enhance phagocytosis, and promote wound healing (Primon-Barros and Macedo, 2017; Abdallnasser Amen et al., 2025). Furthermore, certain peptides exhibit anti-inflammatory properties that are beneficial in the treatment of infectious diseases. Intraperitoneal injections of NDBP-5.5 have been shown to cause substantial decreases in inflammatory lesions associated with mycobacterial infections in lung tissues (Trentini et al., 2017). These peptides also modulate the function of macrophages, which play a critical role in host defense against intracellular pathogens. Treatment of *Mycobacterium abscessus*-infected macrophages with peptides such as NDBP-5.5 reduces the mycobacterial load within macrophages, potentially through the induction of phagolysosomal fusion, increased production of reactive nitrogen and oxygen species, or modulation of cytokine production. This dual ability to target both the infectious agent and the associated inflammatory response represents a significant therapeutic advantage, potentially reducing tissue damage and improving clinical outcomes. Ultimately, the combination of direct antimicrobial and immunomodulatory activities positions these peptides as potential host-directed therapies that could complement conventional antimicrobial agents (Memariani and Memariani, 2025).

### 4.6 Ion Channel blockade

Certain scorpion venom peptides, particularly those belonging to the disulfide-bridged peptide family, may possess the ability to block ion channels in microbial cells. While channel blockade is a known function in eukaryotic systems, its role in bacterial growth inhibition remains largely speculative due to a lack of electrophysiological studies on microorganisms. This mechanism, which is related to the neurotoxic activity of many scorpion venom components, can also contribute to antimicrobial effects (Mendes et al., 2023; Huang et al., 2026). It is important to clarify that activity against mammalian channels is often a toxicological concern (off-target effect) rather than a therapeutic advantage for treating infections. The structural stability provided by disulfide bridges allows these peptides to maintain the conformation necessary for precise ion channel interaction, undermining microbial stability beyond simple physical lysis.

A prominent example is Scorpine, a 75-amino acid peptide containing three disulfide bridges isolated from *Pandinus imperator*. Scorpine exhibits specific potassium channel-blocking activity in addition to its direct antimicrobial properties (Seyfi et al., 2019; López-Giraldo et al., 2023). For disulfide-rich peptides such as Scorpine, potassium-channel-blocking activity (characterized in eukaryotic systems) is hypothesized to perturb microbial ion homeostasis and contribute to antimicrobial effects, though direct microbial electrophysiological validation remains limited. Similarly, Hge36, a Scorpine-like peptide derived from *Hoffmannihadrurus gertschi*, has been shown to display potent potassium channel-blocking activities alongside inhibitory effects against parasites (Flores-Solis et al., 2016). These *in vitro* observations from select scorpine-like peptides raise the hypothesis that potassium-channel interference could represent a shared functional trait within certain scorpion venom peptide families, but broader experimental confirmation across diverse pathogens is still needed.

Beyond potassium, certain scorpion venom peptides can modulate sodium channel activity to achieve antimicrobial outcomes. Ts1, a multifunctional toxin from *Tityus serrulatus*, has demonstrated significant antifungal activity against *Aspergillus nidulans* at micromolar concentrations (Santussi et al., 2017). The antimicrobial mechanism of Ts1 and related peptides likely involves the modulation of ion channel activity in microbial cells, disrupting normal cellular physiology and energy metabolism essential for fungal growth (Shenkarev et al., 2019). Cysteine-containing peptides, such as the Bactridines from *Tityus discrepans*, exhibit antimicrobial activity linked to specific sodium channel modulation rather than general membrane lysis. Bactridines induce sodium ion efflux which can be blocked by sodium channel blockers like amiloride and mibefradil (Harrison et al., 2014). Collectively, these findings highlight that ion channel blockade represents a sophisticated secondary mechanism of action. By targeting essential electrochemical gradients required for microbial survival, these peptides offer a multifunctional approach to pathogen elimination that complements direct membrane lysis. This dual capability potentially reduces the likelihood of resistance while enhancing therapeutic efficacy, marking ion channel modulation as a vital component of scorpion venom pharmacology. However, as the specific bacterial ion channels remain largely unknown, there is an urgent need for further research in this area.

## 5 Therapeutic potential

### 5.1 Advantages over conventional antibiotics

Scorpion venom-derived AMPs offer several distinct advantages over conventional antibiotics, making them attractive candidates for therapeutic development. A primary benefit is their broad-spectrum activity; many scorpion AMPs exhibit efficacy against bacteria, fungi, viruses, and parasites, providing significant potential for multi-indication development (Rincón-Cortés et al., 2022; Rabea et al., 2025). Furthermore, these peptides demonstrate rapid bactericidal action. Unlike conventional antibiotics that often require hours to days to achieve therapeutic effects, AMPs typically kill microbes within minutes, which could reduce the duration of therapy and limit opportunities for resistance development. This low resistance propensity is further bolstered by their mechanism of action; the combination of membrane disruption and potential intracellular multi-targeting makes it statistically difficult for microbes to develop resistance (Utkin et al., 2022). Additionally, scorpion AMPs often exhibit synergy with conventional antibiotics, potentially enabling dose reduction and helping to overcome existing resistance mechanisms (Sani and Separovic, 2016; Rabea et al., 2025). Finally, some scorpion peptides possess anti-biofilm activity, capable of penetrating and disrupting biofilms that are notoriously resistant to conventional antibiotics. Collectively, these attributes position scorpion venom peptides as powerful alternatives or complements to current antimicrobial strategies.

### 5.2 Challenges in clinical development

Despite their therapeutic promise, scorpion venom-derived antimicrobial peptides face several significant challenges that must be addressed for successful clinical translation (Ranasinghe and McManus, 2013; Abdallnasser Amen et al., 2025; Dahiya et al., 2026). A primary concern is toxicity; many antimicrobial peptides exhibit hemolytic activity and cytotoxicity against mammalian cells at therapeutic concentrations, necessitating careful selectivity optimization to ensure the therapeutic window is sufficiently wide for safety (Ranasinghe and McManus, 2013; Fong-Coronado et al., 2024; Panayi et al., 2024). Stability presents another major hurdle, as peptides are susceptible to proteolytic degradation in biological environments, resulting in short half-lives that limit therapeutic efficacy (Utkin et al., 2022; Abdallnasser Amen et al., 2025). Specifically, susceptibility to serum proteases and rapid renal clearance pose significant challenges for systemic administration (Dahiya et al., 2026). Economic factors also impede development; traditional venom extraction yields minute quantities, making large-scale production economically challenging (Nasr et al., 2023), while synthetic production, though feasible, remains more expensive than conventional antibiotic manufacturing (Ortiz et al., 2015). Finally, effective delivery to infection sites, particularly for intracellular pathogens and biofilm-associated infections, requires innovative formulation strategies to ensure the peptides reach their targets in active concentrations (Ebou et al., 2021; Abdallnasser Amen et al., 2025). Overcoming these barriers is essential for realizing the clinical potential of these unique antimicrobial agents.

### 5.3 Strategies for optimization

To overcome the challenges associated with clinical development and enhance the therapeutic potential of scorpion venom-derived AMPs, researchers have developed multiple strategic approaches (Mosaheb et al., 2018; Utkin et al., 2022). SAR studies enable the systematic investigation of the relationship between peptide structure and biological activity, facilitating the rational design of optimized analogs where modification of key residues can enhance antimicrobial potency while reducing toxicity (Mosaheb et al., 2018, Utkin et al., 2022). Sequence truncation strategies, which shorten peptides to their minimal active sequences, can reduce production costs and potentially improve selectivity for microbial over mammalian cells (Utkin et al., 2022; Panayi et al., 2024). To address stability concerns, the incorporation of D-amino acid substitutions enhances proteolytic resistance while often maintaining or improving antimicrobial activity (Utkin et al., 2022). Similarly, cyclization approaches, including head-to-tail or disulfide-mediated cyclization, increase conformational constraint and proteolytic stability, extending the functional half-life of these peptides (Utkin et al., 2022). Hybrid peptide design, which combines structural elements from different antimicrobial peptides, can yield novel compounds with enhanced activity profiles and reduced toxicity (Mosaheb et al., 2018). Finally, advanced delivery systems, such as encapsulation in liposomes, polymeric nanoparticles, or inorganic nanoparticles, protect peptides from degradation, enable targeted delivery to infection sites, and may further reduce off-target toxicity (Ebou et al., 2021, Abdallnasser Amen et al., 2025). Collectively, these optimization strategies represent a multifaceted approach to transforming promising scorpion venom peptides into viable therapeutic agents capable of addressing the growing crisis of antimicrobial resistance. The comparison of several native scorpion venom-derived antimicrobial peptides and their modified analogs is presented in Table 4.

**Table 4:** Comparison of native scorpion venom-derived antimicrobial peptides and their modified analogs.

Sequence-engineered peptide analogs						References
Native peptide			Modified analog			
Native peptide	Native sequence	Baseline bioactivity	Modified analog name	Modified analog sequence	Quantitative changes	
Mucroporin	LFGLIPSLIGGLVSAFK	Effective against Gram-positive bacteria (MCI: 25 μg/mL *S. aureus*; 50 μg/mL for *B. subtilis*); insensitive against Gram negative bacteria (MCI: >100 μg/mL for *E. coli*); No activity against measles, SARS-CoV, or influenza H5N1	Mucroporin-M1	LFRLIKSLIKRLVSAFK	Enhanced activity against Gram-positive bacteria (MCI: 5 μg/mL for *S. aureus*; 25 μg/mL for *B. subtilis*); effective against Gram negative bacteria (MCI: 12.5 μg/mL for *E. coli*); Potent virucidal activity against measles (EC50: 7.15 μg/mL), SARS-CoV (EC50: 14.46 μg/mL), and H5N1 (EC50: 2.10 μg/mL)	Dai et al. (2008), Li et al. (2011), Souza et al. (2023)
Meucin-18	FFGHLFKLATKIIPSLFQ	Potent; MIC against *S. aureus* ~3 μM; High hemolytic activity (near-complete hemolysis at 12.5 μM)	CH-1	FFGRLFKLATKIIPSLFQ	Enhanced, especially against Gram-negative bacteria (up to 16-fold more potent); effective against biofilms; Lower hemolytic toxicity (<20% hemolysis at 12.5 μM) and good *in vivo* safety (e.g., 500 mg/kg dose non-lethal)	Chang et al. (2025)
BmKn2	FIGAIARLLSKIFGKR	Effective against Gram-positive bacteria (MCI: 6.25 μg/mL for *S. aureus*; 12.5 μg/mL for *B. subtilis*); insensitive against Gram negative bacteria (MCI: >100 μg/mL for *E. coli*); High hemolytic activity (HC50 at 17.13 μM)	BmKn2-7	FIKRIARLLRKIF	Enhanced inhibitory activity against both Gram-positive bacteria (MCI: 3.13 μg/mL for *S. aureus*; 6.25 μg/mL for *B. subtilis*) and Gram-negative bacteria (MCI: 6.25–25 μg/mL for *E. coli*); decreased hemolytic activity (HC50 at 90.27 μM)	Cao et al. (2012), Luo et al. (2021)
AamAP1	FLFSLIPHAIGGLISAFK	Has very poor stability in biological environments; Exhibits moderate activity against bacteria (MIC >20 μM) and no fungicidal effect.	GK-19	GFLFKLIPKAIKKLISKFK	The addition of Glycine slightly decreases hydrophobicity and increases helicity (94.74% vs. 72.22% in AamAP1), which dramatically improved stability. It inhibits bacteria at much lower concentrations (MIC: 3–10 μM) and has fungicidal effect on *C. krusei*, *C. albicans* and *C. glabrata* (MIC: 5–10 μM); reduced hemolytic activity and cytotoxicity.	Song et al. (2022)

Formulation-based optimization						Reference
Peptide	Sequence	Baseline bioactivity	Modified name	Formulation strategy	Observed quantitative effects	
Stigmurin/Analogues	S-WT (FFSLIPSLVGGLISAFK) S1 (FFSLIPKLVKGLISAFK) and S2 (FFKLIPKLVKGLISAFK)	The original Stigmurin (S-WT) has weak antimicrobial activity (MIC > 125 μM) against *B. subtilis*. Modified analogues (S1, S2) are potent, with MIC values of 3.5 μM against *B. subtilis*. The analogues (S1 and S2) exhibit significantly increased hemolytic activity and cytotoxicity.	Encapsulated Stigmurin/Analogues	PLA–PEG nanoparticles with encapsulated peptides	The MIC values for the encapsulated peptides remained the same (3.5 μM) as their free counterparts. Encapsulation drastically reduced hemolytic activity and cytotoxicity.	Thakur et al. (2025)

Table 4 documents sequence-modified scorpion-AMP analogs and formulation-based optimization approaches. Comparison between native peptides and their engineered variants reveals consistent SAR lessons. Point-mutation, residue-truncation and hybrid-peptide design can tune net charge, helical content and hydrophobic moment. Increasing positive charge frequently improves antimicrobial potency; fine-tuning hydrophobicity can decouple antimicrobial efficacy from hemolytic toxicity. Some analogs retain the parent peptide’s membrane-disrupting mode, whereas others gain enhanced intracellular targeting or anti-biofilm functions. Formulation strategies (e.g., PLA–PEG nanoparticle encapsulation) do not alter intrinsic peptide sequence or molecular target, yet they improve proteolytic stability, reduce off-target cytotoxicity and optimize tissue delivery. Importantly, optimal modification strategies are peptide-specific; no universal modification rule can be applied to all scorpion AMPs.

## 6 Future directions

Modern omics technologies have revolutionized the discovery and characterization of scorpion venom antimicrobial peptides (Nasr et al., 2023; Solano-Godoy et al., 2025). Transcriptomic analysis of venom glands enables comprehensive cataloging of toxin-encoding genes, including those for antimicrobial peptides (Yacoub et al., 2020; Solano-Godoy et al., 2025), where high-throughput RNA sequencing (RNA-seq) provides quantitative expression data and reveals sequence diversity arising from gene duplication and alternative splicing (Solano-Godoy et al., 2025). Proteomic approaches complement transcriptomic data by confirming peptide expression and identifying post-translational modifications (Nasr et al., 2023; Arcos et al., 2025). Mass spectrometry-based venomics enables detailed profiling of venom composition, while peptidomics specifically targets the peptide fraction (Nasr et al., 2023), and the integration of transcriptomic and proteomic data provides a comprehensive view of the antimicrobial peptide repertoire of individual species (Arcos et al., 2025; Solano-Godoy et al., 2025). Additionally, the growing availability of sequence data has enabled bioinformatics approaches to antimicrobial peptide discovery (Sani and Separovic, 2016; Furtado et al., 2020). Hidden Markov model-based methods can identify potential AMPs in venom gland transcriptomes based on sequence features (Furtado et al., 2020), while machine learning algorithms trained on known antimicrobial peptides can predict antimicrobial activity and selectivity from sequence alone, enabling virtual screening of large datasets (Sani and Separovic, 2016).

Despite promising bioactivity *in vitro*, comprehensive ADMET (absorption, distribution, metabolism, excretion, toxicity) datasets for scorpion-derived AMPs remain scarce. Most natural scorpion AMPs suffer from rapid proteolytic degradation by serum proteases, short systemic half-life, non-specific hemolysis and cytotoxicity, fast renal clearance, and poor oral bioavailability, collectively limiting systemic administration. Systematic ADMET profiling should be incorporated into early-stage pre-clinical evaluation of scorpion AMP leads.

Quantitative structure–activity relationship (QSAR) modelling, molecular dynamics (MD) simulation and AI-based workflows represent powerful complementary tools to accelerate scorpion-AMP research. QSAR models correlate physicochemical descriptors including net charge, hydrophobic moment and helical propensity with measured antimicrobial and hemolytic outputs. MD simulations dynamically visualize peptide-lipid-bilayer interactions, pore-formation processes, and how residue substitutions alter peptide insertion behavior into microbial membranes. Machine-learning and hidden-Markov-model pipelines mine scorpion-gland transcriptomic datasets to predict novel AMP candidates, perform virtual screening for optimized analogues, and forecast peptide toxicity and selectivity before chemical synthesis. Currently, most existing AMP prediction models are trained on general AMP datasets; scorpion-specific fine-tuned AI models remain rare. Integrating multi-omics datasets with computational tools will greatly accelerate the discovery and rational optimization of scorpion-venom-derived antimicrobials.

Advances in synthetic biology offer solutions to the production challenges associated with venom-derived peptides (Abdallnasser Amen et al., 2025; Dahiya et al., 2026). Recombinant expression systems in bacteria, yeast, or plants can produce large quantities of peptides at reduced cost (Dahiya et al., 2026), while fusion protein strategies can enhance expression yields and simplify purification, also masking toxicity to production hosts (Abdallnasser Amen et al., 2025). The future of scorpion venom-derived antimicrobials likely lies in combination with conventional antibiotics or other antimicrobial peptides (Sani and Separovic, 2016; Rabea et al., 2025). Synergistic interactions can enhance efficacy, reduce required doses, and suppress resistance development, where understanding the mechanistic basis of synergy will enable rational design of effective combination regimens (Sani and Separovic, 2016). Finally, the variability in human microbiota and pathogen susceptibility suggests that antimicrobial peptide therapy might benefit from personalized approaches. Rapid diagnostic technologies could enable selection of optimal peptides or combinations based on the specific pathogen profile of individual patients (Sani and Separovic, 2016), ensuring tailored and effective treatment strategies.

## 7 Conclusion

Scorpion venom represents a rich and underexplored source of antimicrobial peptides with significant therapeutic potential. The structural diversity of these molecules, honed through millions of years of evolution, provides a vast library of bioactive compounds with broad-spectrum antimicrobial activity, rapid killing kinetics, and mechanisms of action that circumvent conventional resistance mechanisms. From antibacterial and antifungal to antiviral and antiparasitic applications, scorpion venom peptides demonstrate remarkable versatility.

Despite the challenges of toxicity, stability, production costs, and delivery that currently limit clinical translation, ongoing research is yielding innovative solutions. Rational design strategies, guided by structure–activity relationship studies and enabled by omics technologies, are producing optimized analogs with enhanced therapeutic indices. Advanced delivery systems, including nanoparticle formulations, promise to improve pharmacokinetics and target specificity.

As antimicrobial resistance continues to escalate globally, the need for novel therapeutic agents has never been more urgent. Scorpion venom-derived antimicrobial peptides, empowered by structure–activity-relationship-guided rational design, multi-omics discovery workflows and emerging computational tools including ADMET profiling, molecular dynamics simulation and AI-assisted peptide engineering, have the potential to contribute significantly to the antimicrobial arsenal of the future. Interdisciplinary collaboration among toxicology, peptide chemistry, microbiology, pharmacology, and clinical medicine will be essential to translate the promise of these natural molecules into clinically useful therapies that can combat the growing threat of drug-resistant infections.
